# Outcomes after radical hysterectomy in patients with early-stage adenocarcinoma of uterine cervix

**DOI:** 10.1038/sj.bjc.6605705

**Published:** 2010-06-08

**Authors:** J-Y Park, D-Y Kim, J-H Kim, Y-M Kim, Y-T Kim, J-H Nam

**Affiliations:** 1 Department of Obstetrics and Gynecology, University of Ulsan College of Medicine, Asan Medical Center, No. 388-1 Poongnap-2 dong, Songpa-gu, Seoul 138-736, Korea

**Keywords:** early-stage cervical cancer, squamous cell carcinoma, adenocarcinoma, radical hysterectomy, prognosis

## Abstract

**Background:**

To determine the prognostic factors and treatment outcomes of patients with early-stage adenocarcinoma (AdCa) of uterine cervix who underwent radical hysterectomy (RH).

**Methods:**

Patients with early-stage squamous cell carcinoma (SCCa) of the uterine cervix who underwent RH were compared with patients with AdCa by multivariate analysis.

**Results:**

A total of 1218 patients were eligible, of which 996 (81.8%) had SCCa and 222 (18.2%) had AdCa. In multivariate analysis, parametrial involvement and lymph node metastasis were significant factors for both recurrence-free survival(RFS) and overall survival (OS) of patients with AdCa, whereas age, tumour size, parametrial involvement and lymph node metastasis were significant factors for both RFS and OS of patients with SCCa. After adjusting for significant prognostic factors, patients with AdCa had significantly poorer RFS (odds ratio (OR)=2.07, 95% confidence interval (CI)=1.37–3.12, *P*=0.001) and OS (OR=2.56, 95% CI=1.65–3.96, *P*<0.001) than patients with SCCa. Recurrence outside the pelvis was more frequent in AdCa than in those with SCCa (75 *vs* 57.8%, *P*=0.084).

**Conclusion(s):**

Although RH is still acceptable for treatment of patients with AdCa, a more effective systemic adjuvant therapy is required.

Cervical cancer is the second most common female cancer and the third most common cause of cancer deaths in women worldwide (Parkin *et al*, 2001; Waggoner, 2003). Approximately 75% of cervical cancers are squamous cell carcinomas (SCCas) and 15% are adenocarcinomas (AdCas), and the remainder consists of other rare histologic types (Kosary, 1994; Farley *et al*, 2003). Recently, however, the relative proportion and the absolute incidence of AdCa, compared with SCCa, have increased (Smith *et al*, 2000; Liu *et al*, 2001; Sasieni and Adams, 2001). Nevertheless, there is no uniformly accepted form of management for AdCa. As with SCCa, patients with International Federation of Obstetrics and Gynecology (FIGO) stage IA2–IIA cervical AdCa are treated by radical hysterectomy (RH). The prognosis of patients with AdCa after RH is unclear, primarily because studies have been performed on small numbers of patients. Some of these studies found that patients with AdCa have poorer prognosis than do those with SCCa (Hopkins and Morley, 1991; Eifel *et al*, 1995; Look *et al*, 1996; Samlal *et al*, 1997; Lai *et al*, 1999; Kim *et al*, 2000; Nakanishi *et al*, 2000), whereas other reports found no differences in prognosis (Anton-Culver *et al*, 1992; Miller *et al*, 1993; Shingleton *et al*, 1995; Grisaru *et al*, 2001; Ayhan *et al*, 2004; Lee *et al*, 2006; Fregnani *et al*, 2008; Kasamatsu *et al*, 2009). Therefore, the prognosis after RH and the optimal management of AdCa are still a subject of debate.

The aim of this study was to clarify the treatment outcomes and prognostic factors after RH in patients with FIGO stage IA2–IIA AdCa of uterine cervix, and to compare them with those of patients with SCCa to postulate the optimal management of patients with early-stage AdCa of uterine cervix.

## Materials and methods

From 1989 to 2006, 2350 patients with invasive cervical cancer were treated and followed-up at the Asan Medical Center (AMC, Seoul, Korea). After approval by the Institutional Review Board of AMC, we searched the cancer registry and computerised database of AMC for patients with (1) FIGO stage IA2–IIA cervical cancer, (2) who underwent RH with pelvic and/or para-aortic lymphadenectomy and (3) who had the histologic types of SCCa and AdCa. Patients who received chemotherapy, radiation therapy (RT) or concurrent chemoradiation therapy (CCRT) before RH, and patients with occult cervical cancer detected after simple hysterectomy, were excluded. As the preferred treatment in our centre for patients with FIGO stage IA2–IIA cervical cancer is RH, almost all patients with FIGO stage IA2–IIA cervical cancer underwent RH and only a small number of patients who were not eligible for radical surgery because of severe medical co-morbidity received RT or CCRT. Radical hysterectomy was completed in patients with positive pelvic or para-aortic lymph nodes or parametrial involvement confirmed by frozen section, although it is still unclear whether the surgeon should complete the RH or stop the procedure and administer CCRT if a frozen biopsy reveals parametrial involvement or lymph node metastasis. Patients with >2 intermediate risk factors (lymphovascular space invasion (LVSI), tumour >4 cm or deep cervical stromal invasion >2 out of 3) were recommended for adjuvant RT, whereas patients with one or more high-risk factors (resection margin involvement, parametrial involvement or lymph node involvement) were recommended for adjuvant CCRT. This policy for adjuvant therapy has been consistent during study periods. Medical records were retrospectively reviewed, and the following parameters were collected: age, FIGO stage, histology, grade of differentiation, tumour size, depth of cervical stromal invasion, parametrial involvement, resection margin status, lymphovascular space invasion, lymph node metastasis, adjuvant therapy, date of recurrence, location of recurrent disease, treatment at recurrence, and date of death or last follow-up. Pathologic slides were reviewed by two experienced pathologists at our institution. Squamous cell carcinomas were graded histologically as well-differentiated (grade 1; mature squamous cells with abundant keratin, pearl formation and sometimes intercellular bridges), moderately differentiated (grade 2; less abundant cytoplasm, cell borders less distinct, nuclei with greater pleomorphism and high mitotic activity) and poorly differentiated (grade 3; masses and nests of small, primitive-appearing oval cells with scant cytoplasm and hyperchromatic and spindle-shaped nuclei with high mitotic activity).

All patients were followed-up at least every 3 months for the first 2 years, at least every 6 months for the next 3 years and then every year until recurrence or death. Recurrence-free survival (RFS) was defined as the time, in months, from the date of RH to the date of relapse or censoring, and overall survival (OS) was defined as the time, in months, from the date of RH to the date of death, last follow-up or censoring.

### Statistical analysis

Frequency distributions were compared using the *χ*^2^ and Fisher's exact tests, and mean values were compared between groups using Student's *t*-test. The RFS and OS were estimated using the Kaplan–Meier method and groups were compared by the log-rank test for categorical factors and Cox's proportional hazards model for continuous factors, by univariate analysis. All prognostic variables found to be significant in univariate analysis were included in multivariate analysis using Cox's proportional hazards model. Stepwise backward elimination methods were used to select factors for inclusion in the multivariate Cox proportional hazards model (inclusion criteria, *P*<0.05; exclusion criteria, *P*>0.1). *P*-values <0.05 in two-sided tests were regarded as significant. Data analyses were performed using SPSS for Windows (version 11.0; SPSS Inc., Chicago, IL, USA). Power calculations were performed using NCSS software (version 2004; NCSS Inc., Kaysville, UT, USA). A two-sided log-rank test with an overall sample size of 1218 subjects (222 in group 1 and 996 in group 2) was calculated to achieve 92% power at a 0.05 significance level to detect a difference of 0.08 between 0.82 and 0.90, the proportions surviving in groups 1 and 2, respectively. This corresponded to a hazard ratio of 0.53. The proportion of patients lost during follow-up was 0.05. These results were based on the assumption that the hazard rates were proportional.

## Results

During the study period, 1218 patients met all inclusion criteria. Of these, 996 patients (81.8%) had SCCa and 222 (18.2%) had AdCa. Of the 222 patients with AdCa, 203 had mucinous-type tumours, 14 had endometrioid-type tumours, 2 had serous-type tumours and 3 had mixed-type tumours. The characteristics of these patients are shown in Table 1. Patients with AdCa were significantly younger than those with SCCa. Lymphovascular space invasion was more frequent is patients with AdCa compared with those with SCCa. However, FIGO stage, grade of differentiation, tumour size, depth of cervical stromal invasion, parametrial involvement, resection margin status and lymph node metastasis did not differ between the two histologic groups.

Patients with FIGO stage IA2 disease (*n*=88) underwent type II hysterectomy with pelvic and/or para-aortic lymphadenectomy and patients with FIGO stage IB1–IIA disease (*n*=1130) underwent type III hysterectomy with pelvic and/or para-aortic lymphadenectomy. Thus, all patients underwent pelvic lymphadenectomy, and 731 underwent para-aortic lymphadenectomy. Of the 1218 patients, 311 (25.6%) received postoperative adjuvant therapy. There were no differences in the proportion of patients who received postoperative adjuvant therapy and in the type of adjuvant therapy between the two histologic groups (Table 1).

### Survival and patterns of recurrence in patients with AdCa and SCCa

The overall median and mean follow-up times were 91 and 83 months (range, 10–236 months) for all patients, respectively, 73 and 81 months (range, 11–231 months), respectively, in patients with AdCa and 85 and 92 months (range, 10–236 months), respectively, in patients with SCCa (AdCa *vs* SCCa, *P*=0.012). Of the 1218 patients, 419 had follow-up durations of <60 months, with 137 lost to follow-up for over 1 year at the time of this analysis. Of these, 29 had AdCa and 108 had SCCa, making the rates of loss during follow-up 13.1% (29 out of 222) for the AdCa group, and 10.8% (108 out of 996) for the SCCa group (*P*=0.344). The mean and median follow-up times of patients lost to follow-up were 41 and 43 months (range, 14–59 months), respectively, for the AdCa group, and 43 and 41 months (range, 10–59 months), respectively, for the SCCa group (*P*=0.440). The 5-year and 10-year RFS rates in patients with AdCa were 86 and 82%, respectively, and the 5-year and 10-year RFS rates in patients with SCCa were 92 and 90%, respectively (*P*=0.009). The 5-year and 10-year OS rates in patients with AdCa were 90 and 83%, respectively, and the 5-year and 10-year OS rates in patients with SCCa were 94 and 91%, respectively (*P*<0.001). At the time of this analysis, 32 patients (14.4%) in AdCa group and 90 patients (9.0%) in SCCa group had cancer recurrence (*P*=0.016), and 30 patients (13.5%) in AdCa group and 71 patients (7.1%) in SCCa died of disease (*P*=0.002). The mean times to recurrence in the AdCa and SCCa groups were 36 months (range, 6–152 months) and 34 months (range, 2–156 months), respectively (*P*=0.805). The anatomic location of tumour at first recurrence was outside the pelvis in 57.8 and 75.0% of patients with SCCa and AdCa, respectively (*P*=0.084) (Table 2). Recurrent tumours in these patients were observed in the abdomen, skin, muscle, bone, liver, lung, meninx, brain and/or lymph nodes.

The treatment modality or strategy was not altered over time. Therefore, we divided the study period arbitrarily into three 6-year intervals (1989–1994, *n*=156; 1995–2000, *n*=371; and 2001–2006, *n*=691). However, we found that 5-year disease-free survival rates (95, 92 and 90%, respectively, *P*=0.1425) and 5-year OS rates (97, 94 and 92%, respectively, *P*=0.1889) did not differ by time.

### Prognostic factors associated with RFS and OS in patients with AdCa and SCCa

In 222 patients with AdCa, FIGO stage, tumour size, depth of cervical stromal invasion, parametrial involvement, resection margin status, LVSI, lymph node metastasis and type of adjuvant treatment were significantly associated with both RFS and OS in univariate analysis, whereas age, grade of differentiation and histologic subtype were unrelated (Table 3). After adjusting for factors significant in univariate analysis, multivariate analysis showed that parametrial involvement and lymph node metastasis were significant factors for both RFS and OS (Table 4).

In 996 patients with SCCa, age, tumour size, depth of cervical stromal invasion, parametrial involvement, LVSI, lymph node metastasis and type of adjuvant treatment were significantly associated with both RFS and OS, but FIGO stage was significantly associated with only RFS in univariate analysis, whereas resection margin status and grade of differentiation were unrelated (Table 5). After adjusting for factors significant in univariate analysis, multivariate analysis showed that age, tumour size, parametrial involvement and lymph node metastasis were significant factors for both RFS and OS (Table 6).

When analysing 1218 patients with AdCa and SCCa, age, FIGO stage, histologic type, tumour size, depth of cervical stromal invasion, parametrial involvement, LVSI, lymph node metastasis and type of adjuvant treatment were significantly associated with both RFS and OS, whereas resection margin status and grade of differentiation were unrelated (Table 7). After adjusting for factors significant in univariate analysis, multivariate analysis showed that age, histologhic type, tumour size, parametrial involvement and lymph node metastasis were significant factors for both RFS and OS (Table 8). Relative to patients with SCCa, the probability of cancer recurrence was significantly higher in patients with AdCa (odds ratio (OR)=2.07, 95% confidence interval (CI)=1.37–3.12, *P*=0.001) after adjusting factors significant in univariate analysis. Moreover, the probability of cancer death was significantly higher in patients with AdCa (OR=2.56, 95% CI=1.65–3.96, *P*<0.001) compared with patients with SCCa after adjusting factors significant in univariate analysis.

## Discussion

In our series, the survival outcomes after RH followed by tailored adjuvant therapy according to postoperative risk factors in patients with IA2–IIA AdCa of uterine cervix were excellent because 5-year RFS and OS were 86 and 90%, respectively. Parametrial involvement and lymph node metastasis were independent prognostic factors in patients with AdCa as were they in patients with SCCa. Therefore, this treatment strategy for IA2–IIA AdCa of uterine cervix seems acceptable as with SCCa. However, in spite of the distribution of postoperative risk factors, the proportion of patients who received adjuvant therapy, and type of adjuvant therapy were not different between AdCa and SCCa groups, the RFS and OS were significantly poorer in AdCa group compared with SCCa group in multivariate analysis although the survival differences were not much. The time interval to recurrence was not different between AdCa and SCCa groups, but recurrence outside pelvis was more frequent in AdCa groups. This may suggest that AdCa has somewhat aggressive behaviour with propensity of distant metastasis and systemic adjuvant therapy might be beneficial for AdCa.

It has long been unclear whether prognosis of patients with early-stage cervical cancer undergoing RH was dependent on histologic type. Although some studies included a significant number of subjects, most previous reports had small cohorts of patients, with numbers insufficient to determine small differences in RFS and OS. Hence, because the survival of surgically treated patients with early-stage cervical cancer is excellent, the magnitude of differences in RFS and OS among different histologic types is small. Previous studies have reported that the magnitude of differences in 5-year RFS and OS rates ranged from 2.0 to 9.0%, findings similar to ours (Look *et al*, 1996; Lai *et al*, 1999; Nakanishi *et al*, 2000; Ayhan *et al*, 2004; Fregnani *et al*, 2008). As far as we know, our series is one of the largest studies which compared the survival outcomes of early-stage AdCa and SCCa. The survival difference between AdCa and SCCa groups was small but significant. In our series, a two-sided log-rank test with an overall sample size of 1218 subjects (of which 222 are in AdCa group and 996 are in SCCa group) achieved 80% power at a 0.05 significance level to detect a difference of 6% in 10-year DFS between 86 and 92%, the proportions surviving in AdCa group and SCCa group, respectively. A two-sided log-rank test with an overall sample size of 1218 subjects (of which 222 are in AdCa group and 996 are in SCCa group) achieved 92% power at a 0.05 significance level to detect a difference of 8% in 10-year OS between 82 and 90%, the proportions surviving in AdCa group and SCCa group, respectively.

Although we found that patients with AdCa had significantly poorer prognosis than those with SCCa, after adjusting for other significant prognostic factors, the magnitude of differences in RFS and OS were small and the RFS and OS of patients with AdCa and AdSCCa were still good. Therefore, the current management strategy for patients with early-stage AdCa, consisting of RH followed tailored adjuvant therapy according to postoperative risk factors, should be acceptable.

Of the 20 patients with AdCas 4–6 cm in diameter, 4 had recurrent disease and 3 of these died of disease within 5 years, making the 5-year DFS and OS rates 77 and 90%, respectively. The fourth patient with recurrence died of disease at 78 months, making the survival rate at 78 months 80%, similar to the 40–70% OS rates previously reported.

It is still not clear whether histologic type has an effect on time to recurrence after RH, or on the patterns of spread and recurrence. Although several studies reported that the time to recurrence was shorter in patients with AdCa and AdSCCa than in those with SCCa, more recent work has found no difference associated with histologic type (Look *et al*, 1996; Lai *et al*, 1999). When classified into recurrence inside and outside the pelvis, the pattern of spread and recurrence did not differ among histologic groups in some studies (Look *et al*, 1996; Lai *et al*, 1999; Grisaru *et al*, 2001), whereas other reports showed an association between histologic type and more frequent disseminated peritoneal spread or distant metastasis (Drescher *et al*, 1989; Eifel *et al*, 1995; Kasamatsu *et al*, 2009). In our series, recurrence outside the pelvis was more frequent in patients with AdCa. As a result of the absence of curative systemic therapy, patients with recurrence outside the pelvis tend to perform very poorly; however, patients with isolated pelvic recurrence are frequently salvaged with pelvic RT with or without concurrent chemotherapy. This may be one reason for the differences in OS observed among histologic types. Therefore, to improve the survival of patients with AdCa after RH, more effective systemic adjuvant therapies are required.

Consistently reported independent adverse prognostic factors for patients with early-stage cervical cancer after RH include higher FIGO stage, lymph node metastasis, parametrial involvement, depth of cervical stromal invasion, tumour size and LVSI (Look *et al*, 1996; Lai *et al*, 2007; Kasamatsu *et al*, 2009). We also found that these factors were significant in univariate analysis, although only lymph node metastasis, parametrial involvement and tumour size were significant in multivariate analysis. Some investigators reported that AdCa was more frequently associated with these prognostic factors than SCCa (Kasamatsu *et al*, 2009). However, other studies found no significant association between these factors and histologic type (Shingleton *et al*, 1995; Look *et al*, 1996; Nakanishi *et al*, 2000; Fregnani *et al*, 2008). In our series, there were no differences in FIGO stage, tumour size, depth of cervical stromal invasion, parametrial involvement, LVSI or lymph node metastasis between patients with the two histologic types.

In conclusion, we found that the clinicopathologic characteristics and time interval to recurrence did not differ between histologic types. However, recurrence outside the pelvis was more frequent in patients with AdCa than in those with SCCa. Moreover, patients with AdCa had significantly poorer RFS and OS than did those with SCCa. Nevertheless, the current treatment strategy of RH on patients with AdCa is acceptable because the RFS and OS were still excellent. However, more effective systemic adjuvant therapies after RH are needed for patients with AdCa.

## Figures and Tables

**Table 1:** Characteristics of the study patients (*n*=1218)

		**AdCa**	**SCCa**	
**Characteristics**	**Total**	**(*n*=222)**	**(*n*=996)**	***P*-value**
*Age, years*				
Mean (range)	48.2 (24–86)	46.4 (25–73)	48.7 (24–86)	0.006
<30	21 (1.7%)	5 (2.3%)	16 (1.6%)	0.177
30–49	715 (58.7%)	141 (63.5%)	574 (57.6%)	
>50	482 (39.6%)	76 (34.2%)	406 (40.8%)	
				
*FIGO stage*				
IA2	88 (7.2%)	8 (3.6%)	80 (8.0%)	0.061
IB1–IB2	1019 (83.7%)	195 (87.8%)	824 (82.7%)	
IIA	111 (9.1%)	19 (8.6%)	92 (9.3%)	
				
*Histologic subtype*				
Mucinous	203 (16.7%)	203 (16.7%)	—	—
Endometrioid	14 (1.1%)	14 (1.1%)	—	
Serou	2 (0.2%)	2 (0.2%)	—	
Mixed	3 (0.2%)	3 (0.2%)	—	
				
*Grade of differentiation*				
Well	332 (27.3%)	63 (28.4%)	269 (27.0%)	0.366
Moderately	573 (47.0%)	94 (42.3%)	479 (48.1%)	
Poorly	294 (24.1%)	60 (27.0%)	234 (23.5%)	
Undetermined	19 (1.6%)	5 (2.3%)	14 (1.4%)	
				
*Tumour size, cm*				
Mean (range)	2.4 (0.3–9)	2.3 (0.3–6)	2.4 (0.4–9)	0.286
⩽2	571 (46.9%)	108 (48.6%)	463 (46.5%)	0.147
2–4	510 (41.9%)	94 (42.3%)	416 (41.8%)	
4–6	114 (9.4%)	20 (9.0%)	94 (9.4%)	
>6	23 (1.9%)	0. (0.0%)	23 (2.3%)	
				
*Depth of stromal invasion*				
⩽1/2	753 (61.8%)	135 (60.8%)	618 (62.0%)	0.731
>1/2	465 (38.2%)	87 (39.2%)	378 (38.0%)	
				
*Parametrial involvement*				
No	1106 (90.8%)	200 (90.1%)	906 (91.0%)	0.684
Yes	112 (9.2%)	22 (9.9%)	90 (9.0%)	
				
*Resection margin*				
Negative	1190 (97.7%)	218 (98.2%)	972 (97.6%)	0.585
Positive	28 (2.2%)	4 (1.8%)	24 (2.4%)	
				
*LVSI*				
No	969 (79.6%)	189 (85.1%)	780 (78.3%)	0.023
Yes	249 (20.4%)	33 (14.9%)	216 (21.7%)	
				
*Lymph node metastasis*				
No	1048 (86.0%)	188 (84.7%)	860 (86.3%)	0.518
Yes	170 (14.0%)	34 (15.3%)	136 (13.7%)	
				
*PALN metastasis* a				
No	1204 (98.9%)	219 (98.6%)	985 (98.9%)	0.755
Yes	14 (1.1%)	3 (1.4%)	11 (1.1%)	
				
*Adjuvant treatment*				
None	907 (74.5%)	159 (71.6%)	748 (75.1%)	0.242
Chemotherapy	86 (7.1%)	20 (9.0%)	66 (6.6%)	
Radiation therapy	113 (9.3%)	26 (11.7%)	87 (8.7%)	
CCRT	112 (9.2%)	17 (7.7%)	95 (9.5%)	

Abbreviations: AdCa=adenocarcinoma; CCRT=concurrent chemoradiation therapy; FIGO=International Federation of Obstetrics and Gynecology; LVSI=lymphovascular space invasion; PALN=para-aortic lymph node; SCCa=squamous cell carcinoma.

a A total of 731 patients underwent para-aortic lymphadenectomy.

**Table 2:** The anatomic location of tumour at first recurrence by histologic type of tumour (*N*=122)

	**Histologic type**				
**Location of tumour**	**SCCa**	**AdCa**	**Total**		
Pelvis	38 (42.2%)	8 (25.0%)	46 (37.7%)		
					
*Outside pelvis*	62 (67.8%)	24 (75.0%)	76 (62.3%)		
Abdomen^a^	1 (1.1%)	7 (21.9%)	8 (6.6%)		
Distant metastasis^b^	42 (46.7%)	11 (34.4%)	53 (43.4%)		
Pelvis and abdomen	3 (3.3%)	4 (12.5%)	7 (5.7%)		
Pelvis and distant metastasis	3 (3.3%)	2 (6.3%)	5 (4.1%)		
Abdomen and distant metastasis	2 (2.2%)	0 (0%)	2 (1.6%)		
Pelvis, abdomen and distant metastasis	1 (1.1%)	0 (0%)	1 (0.8%)		
Total	90 (100.0%)	32 (100.0%)	122 (100.0%)		

Abbreviations: AdCa=adenocarcinoma; SCCa=squamous cell carcinoma.

a Abdominal peritoneal metastasis.

b Haematogenous metastasis to extraperitoneal organs.

**Table 3:** Univariate analyses of clinicopathologic parameters on recurrence-free and overall survival in patients with adenocarcinoma

	**Adenocarcinoma (*n*=222)**				
		**Recurrence-free survival**		**Overall survival**	
**Variables**	***N* (%)**	**5-year rate (%)**	***P*-value**	**5-year rate (%)**	***P*-value**
*Age, years*					
As continuous variable	222 (100)		0.153		0.073
<30	5 (2.3)	80	0.250	80	0.096
30–49	141 (63.5)	89		93	
>50	76 (34.2)	81		86	
					
*FIGO stage*					
IA2	8 (3.6%)	100	<0.001	100	<0.001
IB1–IB2	195 (87.8%)	89		92	
IIA	19 (8.6%)	52		65	
					
*Histologic subtype*					
Mucinous	203 (16.7%)	87	0.373	90	0.425
Endometrioid	14 (1.1%)	77		84	
Serou	2 (0.2%)	50		50	
Mixed	3 (0.2%)	100		100	
					
*Grade of differentiation* a					
Well	63 (29.0)	88	0.688	90	0.532
Moderately	94 (43.3)	83		91	
Poorly	60 (27.6)	87		89	
					
*Tumour size, cm*					
⩽2	108 (48.6)	91	0.041	95	0.033
2–4	94 (42.3)	82		85	
4–6	20 (9.0)	77		90	
>6	0 (0.0)	—		—	
					
*Depth of stromal invasion*					
⩽1/2	135 (60.8)	92	0.002	94	0.008
>1/2	87 (39.2)	78		84	
					
*Parametrial involvement*					
No	200 (90.1)	89	<0.001	92	<0.001
Yes	22 (9.9)	55		66	
					
*Resection margin*					
Negative	218 (98.2)	87	0.013	91	0.005
Positive	4 (1.8)	38		38	
					
*LVSI*					
No	189 (85.1)	88	0.033	92	0.026
Yes	33 (14.9)	75		80	
					
*Lymph node metastasis*					
No	188 (84.7)	90	<0.001	94	<0.001
Yes	34 (15.3)	66		69	
					
*Adjuvant treatment*					
None	159 (71.6)	89	0.003	94	<0.001
Chemotherapy	20 (9.0)	88		95	
Radiation therapy	26 (11.7)	76		77	
CCRT	11 (7.7)	71		70	

Abbreviations: CCRT=concurrent chemoradiation therapy; FIGO=International Federation of Obstetrics and Gynecology; LVSI=lymphovascular space invasion.

a Grade of differentiation was undetermined in 21 patients.

**Table 4:** Univariate analyses of clinicopathologic parameters on recurrence-free and overall survival in patients with squamous cell carcinoma

	**Squamous cell carcinoma (*n*=996)**				
		**Recurrence-free survival**		**Overall survival**	
**Variables**	***N***	**5-year rate (%)**	***P*-value**	**5-year rate (%)**	***P*-value**
*Age, years*					
As continuous variable	996 (100)		<0.001		<0.001
<30	16 (1.6)	94	<0.001	94	<0.001
30–49	574 (57.6)	94		97	
>50	406 (40.8)	88		91	
					
*FIGO stage*					
IA2	80 (8.0)	100	0.002	100	0.061
IB1–IB2	824 (82.7)	92		94	
IIA	92 (9.3)	83		91	
					
*Grade of differentiation* a					
Well	269 (27.4)	92	0.705	93	0.896
Moderately	479 (48.8)	92		94	
Poorly	234 (23.8)	90		93	
					
*Tumour size, cm*					
⩽2	463 (46.5)	97	<0.001	98	<0.001
2–4	416 (41.8)	89		92	
4–6	94 (9.4)	80		87	
>6	23 (2.3)	75		77	
					
*Depth of stromal invasion*					
⩽1/2	618 (62.0)	94	<0.001	95	0.001
>1/2	378 (38.0)	87		91	
					
*Parametrial involvement*					
No	906 (91.0)	93	<0.001	95	<0.001
Yes	90 (9.0)	78		82	
					
*Resection margin*					
Negative	972 (97.6)	92	0.399	94	0.170
Positive	24 (2.4)	96		100	
					
*LVSI*					
No	780 (78.3)	93	<0.001	95	0.003
Yes	216 (21.7)	87		90	
					
*Lymph node metastasis*					
No	860 (86.3)	93	<0.001	95	<0.001
Yes	136 (13.7)	82		85	
					
*Adjuvant treatment*					
None	748 (75.1)	94	<0.001	96	<0.001
Chemotherapy	66 (6.6)	89		91	
Radiation therapy	87 (8.7)	84		87	
CCRT	95 (9.5)	82		85	

Abbreviations: CCRT=concurrent chemoradiation therapy; FIGO=International Federation of Obstetrics and Gynecology; LVSI, lymphovascular space invasion.

a Grade of differentiation was undetermined in 21 patients.

**Table 5:** Multivariate analyses of clinicopathologic parameters on recurrence-free and overall survival in patients with adenocarcinoma

	**Adenocarcinoma (*n*=222)**				
		**Recurrence-free survival^a^**		**Overall survival^a^**	
**Variables**	***N***	**OR (95% CI)**	***P*-value**	**OR (95% CI)**	***P*-value**
*Parametrial involvement*					
No	200 (90.1)	Reference		Reference	
Yes	22 (9.9)	3.46 (1.46–8.19)	0.005	6.17 (2.55–14.95)	0.002
					
*Lymph node metastasis*					
No	188 (84.7)	Reference		Reference	
Yes	34 (15.3)	2.45 (1.14–5.30)	0.022	2.79 (1.25–6.25)	0.012

Abbreviations: CI=confidence interval; LVSI=lymphovascular space invasion; OR=odds ratio.

a The analysis included FIGO stage, tumour size, depth of stromal invasion, parametrial involvement, resection margin, LVSI, lymph node metastasis, and adjuvant treatment.

**Table 6:** Multivariate analyses of clinicopathologic parameters on recurrence-free and overall survival in patients with squamous cell carcinoma

	**Squamous cell carcinoma (*n*=996)**				
		**Recurrence-free survival^a^**		**Overall survival^b^**	
**Variables**	***N***	**OR (95% CI)**	***P*-value**	**OR (95% CI)**	***P*-value**
*Age, years*					
As continuous variable	996 (100)	1.05 (1.03–1.07)	<0.001	1.05 (1.03–1.07)	<0.001
					
*Tumour size, cm*					
⩽2	463 (46.5)	Reference		Reference	
2–4	416 (41.8)	2.25 (1.30–3.89)	0.004	2.12 (1.16–3.88)	0.015
4–6	94 (9.4)	4.51 (2.32–8.78)	<0.001	3.51 (1.64–7.50)	0.001
>6	23 (2.3)	6.36 (2.43–16.68)	<0.001	5.11 (1.73–15.14)	0.003
					
*Parametrial involvement*					
No	906 (91.0)	Reference		Reference	
Yes	90 (9.0)	1.78 (1.01–3.12)	0.046	2.17 (1.13–4.13)	0.019
					
*Lymph node metastasis*					
No	860 (86.3)	Reference		Reference	
Yes	136 (13.7)	1.86 (1.12–3.09)	0.017	2.11 (1.18–3.75)	0.012

Abbreviations: CI=confidence interval; LVSI=lymphovascular space invasion; OR=odds ratio.

a The analysis included age, FIGO stage, tumour size, depth of stromal invasion, parametrial involvement, LVSI, lymph node metastasis and adjuvant treatment.

b The analysis included age, tumour size, depth of stromal invasion, parametrial involvement, LVSI, lymph node metastasis and adjuvant treatment.

**Table 7:** Univariate analyses of clinicopathologic parameters on recurrence-free and overall survival in all patients (*n*=1218)

		**Recurrence-free survival**		**Overall survival**	
**Variables**	**No. of patients**	**5-year rate (%)**	***P*-value**	**5-year rate (%)**	***P*-value**
*Age, years*					
As continuous variable	1218 (100%)		<0.001		<0.001
<30	21 (1.7%)	90	<0.001	90	<0.001
30–49	715 (58.7%)	93		96	
>50	482 (39.6%)	87		90	
					
*FIGO stage*					
IA2	88 (7.2%)	100	<0.001	100	<0.001
IB1–IB2	1019 (83.7%)	91		93	
IIA	111 (9.1%)	80		86	
					
*Histology*					
SCCa	996 (81.8%)	92	0.008	94	<0.001
AdCa	222 (18.2%)	86		90	
					
*Grade of differentiation* a					
Well	332 (27.3%)	91	0.902	93	0.967
Moderately	573 (47.0%)	91		94	
Poorly	294 (24.1%)	89		92	
					
*Tumour size, cm*					
⩽2	571 (46.9%)	96	<0.001	97	<0.001
2–4	510 (41.9%)	88		91	
4–6	114 (9.4%)	80		87	
> 6	23 (1.9%)	75		77	
					
*Depth of stromal invasion*					
⩽1 out of 2	753 (61.8%)	94	<0.001	95	<0.001
>1 out of 2	465 (38.2%)	85		90	
					
*Parametrial involvement*					
No	1106 (90.8%)	92	<0.001	95	<0.001
Yes	112 (9.2%)	74		79	
					
*Resection margin*					
Negative	1190 (97.7%)	91	0.911	93	0.803
Positive	28 (2.2%)	89		93	
					
*LVSI*					
No	1048 (86.0%)	92	<0.001	94	<0.001
Yes	170 (14.0%)	85		89	
					
*Lymph node metastasis*					
No	1111 (85.5%)	93	<0.001	95	<0.001
Yes	188 (14.5%)	78		82	
					
*Adjuvant treatment*					
None	907 (74.5%)	93	<0.001	95	<0.001
Chemotherapy	86 (7.1%)	89		92	
Radiation therapy	113 (9.3%)	82		85	
CCRT	112 (9.2%)	80		83	

Abbreviations: AdCa=adenocarcinoma; CCRT=concurrent chemoradiation therapy; FIGO=International Federation of Obstetrics and Gynecology; LVSI=lymphovascular space invasion; SCCa=squamous cell carcinoma.

a Grade of differentiation was undetermined in 21 patients.

**Table 8:** Multivariate analyses of clinicopathologic parameters on recurrence-free and overall survival in all patients (*n*=1218)

		**Recurrence-free survival^a^**		**Overall survival^a^**	
**Variables**	**No. of patients**	**OR (95% CI)**	***P*-value**	**OR (95% CI)**	***P*-value**
*Age, years*					
As continuous variable	1218 (100%)	1.04 (1.03–1.06)	<0.001	1.05 (1.03–1.06)	<0.001
					
*Histology*					
SCCa	996 (81.8%)	Reference		Reference	
AdCa	222 (18.2%)	2.07 (1.37–3.12)	0.001	2.56 (1.65–3.96)	<0.001
					
*Tumour size, cm*					
⩽2	571 (46.9%)	Reference		Reference	
2–4	510 (41.9%)	2.11 (1.34–3.31)	0.001	2.06 (1.27–3.37)	0.004
4–6	114 (9.4%)	3.55 (2.00–6.31)	<0.001	3.02 (1.59–5.72)	0.001
>6	23 (1.9%)	5.48 (2.17–13.81)	<0.001	4.60 (1.64–12.93)	0.004
					
*Parametrial involvement*					
No	1106 (90.8%)	Reference		Reference	
Yes	112 (9.2%)	2.04 (1.27–3.29)	0.003	2.15 (1.25–3.70)	0.005
					
*Lymph node metastasis*					
No	1111 (85.5%)	Reference		Reference	
Yes	188 (14.5%)	2.07 (1.35–3.18)	0.001	2.27 (1.41–3.64)	0.001

Abbreviations: AdCa, adenocarcinoma; CI, confidence interval; OR, odds ratio; LVSI=lymphovascular space invasion; SCCa, squamous cell carcinoma;

a The analysis included age, FIGO stage, histology, tumour size, depth of stromal invasion, parametrial involvement, resection margin, LVSI, lymph node metastasis and adjuvant treatment.
